# A Project-Management Governance Model for Strengthening Biosafety Oversight in High-Risk Biological Research Facilities Across the United States: A Narrative Review

**DOI:** 10.7759/cureus.100512

**Published:** 2025-12-31

**Authors:** Amienye Omo Enabulele, Bright P Saah, Kwesi Akonu Adom Mensah Forson, Patience A Kwara, Aliyu O Olaniyi

**Affiliations:** 1 College of Business, Missouri State University, Springfield, USA; 2 Civil and Environmental Engineering, Lehigh University, Bethlehem, USA; 3 Biology, University of Virginia, Charlottesville, USA; 4 Nanoscience, University of North Carolina, Greensboro, USA; 5 Geriatrics, Stepping Hill Hospital, Manchester, GBR

**Keywords:** biological research facilities, biosafety oversight, biosecurity, governance model, high-risk laboratories, oversight policy, project management, risk management

## Abstract

In the United States, there are high-risk biological research facilities with complex and overlapping management structures in an attempt to combat accidental or intentional exposure to dangerous pathogens. Even with numerous regulatory mechanisms and institutions biosafety committees, the recent evaluation of biosafety management shows that there are still a lot of gaps in governance, accountability fragmentation and inconsistency in the enforcement of biosafety management practices. The process of enhancing these oversight mechanisms needs to be organized, which is based on uniting the operational, institutional, and federal duties and control under a common system of governance.

This narrative review will suggest a project-management governance model that is more integrative to facilitate better oversight of biosafety in the case of high-risk biological research in the United States. The model aims at enhancing stakeholder coordination, accountability and transparency in biosafety management by implementing project-management concepts in biosafety governance.

A focused literature search was undertaken to locate peer-reviewed articles, governmental documents, and policy brochures within the past five years (2020-2025) with the help of PubMed, Scopus, and Google Scholar being used. Literature covering the issue of biosafety oversight, biosecurity governance and project-management systems were captured. The data were compiled in terms on the theme in order to determine governance aspects that are flexible to manage biosafety. The proposed model was informed by comparison of key concepts in the Project Management Body of Knowledge (PMBOK), ISO 21500, and the rest of governance standards to existing biosafety and biosecurity standards.

The review reported four consistent challenges associated with biosafety governance, including: (1) lack of inter-agency coordination; (2) weak institutional accountability; (3) failure to communicate risk and performance reports/monitoring; and (4) lack of standard projects-governance organization. Project-management principles, which include the stakeholder mapping, the risk registers, the performance measures and the governance boards, can be incorporated to reinforce decision making and accountability. The model suggested is based on the three levels of governance consisting of strategic, operational, and project layer of governance, and the initiative of keeping the feedback and performance loop cycles.

The implementation of the bio-safety regulation with the help of the project-management governance framework is an organized way to integrate the policy implementation, improve the risk treatment, and promote ongoing improvement in the high-risk researches. Put differently, the implementation of this model can strengthen national resiliency in biosafety, facilitate inter-agency partnerships, and develop an organizational culture of accountability and safety in the field of biological research.

## Introduction and background

Context and importance

The United States of America has high-risk biological research centers, especially Biosafety Level-3 (BSL-3) and BSL-4 laboratories, which are high-containment facilities designed to handle pathogens capable of causing serious or fatal disease and requiring progressively stringent engineering and procedural controls, where research on pathogens that may be a threat to the public, agriculture, and national security is conducted [1]. These plants are regulated by several federal organizations such as Centers of Disease Control and Prevention (CDC), the National Institutes of Health (NIH), and the United States Department of Agriculture (USDA), institutional biosafety committees which are under federal direction. These procedures are meant to make sure that the biosafety standards are met, the protection of the personnel and communities are not compromised, as well as the scientific integrity are upheld [2].

Although such good programs are in place, the management of biosafety in risky laboratories is complicated and usually disjointed. The differences in the interpretation of regulation, the lack of uniformity in the application of policies, and the duplication of their roles enhance inefficiencies in the management of risks [2]. More so, the blistering development of biotechnology including gain-of-function research (experimental studies that intentionally enhance biological characteristics such as transmissibility or pathogenicity), gene editing, and synthetic biology are putting current structures of oversight to the task, again highlighting the necessity of adaptive and integrated control systems.

Gap in knowledge

The existing regulation mechanisms are focused more on regulatory compliance, that is, a compliance-based oversight model primarily oriented toward meeting prescribed regulatory requirements rather than continuous, performance-driven risk management. They have the requirement to follow the safety protocols as well as reporting of incidents but, in most cases, lack the system that ensures constant evaluation of performance and interaction with the stakeholders and accountability among the various levels of the organization. This has led to inconsistent quality of oversight, poor inter-agency coordination and inefficient near-miss event learning mechanisms, due to the lack of standardized processes of governance. This problem creates a significant sense of urgency on the creation and deployment of governance models that can go beyond compliance checklists in order to focus on coordination, accountability, and dynamic risk management approaches.

For example, a single high-containment research laboratory may be subject to overlapping oversight from multiple federal agencies and institutional committees, each with different reporting timelines, inspection criteria, and risk-assessment expectations. This fragmentation can lead to unclear accountability, duplicated audits, and delayed communication of near-miss incidents.

Purpose of this narrative review

This literature review aims to integrate literature on the topic of biosafety governance, organizational risk management and project-management theory in a manner that can allow implementing a universal governing structure of biosafety control in the United States. It involves applying project-management governance principles, including makes sense, stakeholder mapping, and continuous improvement cycles, so that this review can create a model which improves coordination and effective oversight in high-risk biological research settings. The suggested framework offers a strategic direction on how to align regulatory goals to operational practices, hence establishing a culture of safety, transparency and shared responsibility in all levels of the biosafety governance.

## Review

Methods

Search Strategy

A targeted literature search was conducted between January and March 2025 using three primary databases: PubMed, Scopus, and Google Scholar. Search terms included combinations of “biosafety oversight,” “biosafety governance,” “biosecurity policy,” “high-risk research facilities,” “project management,” “governance framework,” and “risk management in laboratories.” Boolean operators (AND/OR) were used to combine terms, and searches were iteratively refined across databases to improve relevance and account for differences in indexing. In addition to peer-reviewed articles, relevant policy documents, governmental reports, and governance frameworks were identified through reference list screening and targeted searches of authoritative institutional sources. The search focused on literature published between 2000 and 2025 to capture both foundational and recent developments in biosafety governance.

Inclusion Rationale

Literature was included based on its relevance to biosafety and biosecurity oversight in the United States, with secondary consideration given to international governance models offering transferable lessons for high-risk research environments. Sources addressing organizational governance, project-management frameworks (e.g., Project Management Body of Knowledge (PMBOK), PRINCE2, ISO 21500), and public-sector oversight were included where they provided conceptual insights applicable to biosafety governance. Inclusion was guided by topical relevance, credibility of the source, and contribution to understanding coordination, accountability, and risk management in complex oversight systems.

Selection Process

Titles and abstracts were initially screened to exclude sources that were overly technical, unrelated to governance, or not applicable to biosafety or comparable high-risk environments. Full-text review was then conducted for potentially relevant sources to assess their contribution to biosafety oversight, accountability mechanisms, and governance structures. Study selection was carried out collaboratively by the authors, and inclusion decisions were based on consensus. Any differences in interpretation or relevance were resolved through discussion to ensure alignment with the objectives of the review.

Analytic Approach

A thematic synthesis approach was employed to analyze and integrate the selected literature. A formal risk-of-bias assessment tool was not applied due to the heterogeneity of included sources, which comprised peer-reviewed studies, policy documents, and governance frameworks. Instead, sources were critically appraised based on credibility, policy relevance, methodological rigor where applicable, and consistency with established biosafety and governance principles. Key themes, such as accountability, inter-agency coordination, risk management, performance monitoring, and continuous improvement, were identified through iterative reading and informal coding. These themes were then cross-mapped to established project-management governance frameworks to inform the development of the proposed biosafety governance model.

Review findings and discussion

The State of Biosafety Oversight in the United States

The biosafety regulation in the United States operates in a multilayered control system wherein the federal, state, and institutional governments begin to engage. At the federal level, the USDA and the CDC collaboratively implement the Federal Select Agent Program that regulates the handling and utilization of the select agents and toxins. The NIH Guidelines on Research Involving Recombinant or Synthetic Nucleic Acid Molecules are guided by the NIH Office of Science Policy and it is the primary directive of institutional biosafety committees. Every research institution that undertakes high risk biological research should have a biosafety committee with a responsibility of having their protocols looked at, adherence to the regulations and supervision of laboratory activities [3].

Recent updates from the National Science Advisory Board for Biosecurity (NSABB) in 2024 further emphasize the need for modernized oversight approaches for emerging biotechnologies, particularly in relation to dual-use research and enhanced pathogen potential, reinforcing concerns about fragmentation and accountability in the current system [4]. Similarly, recent White House Office of Science and Technology Policy (OSTP) guidance on biotechnology and biosecurity governance underscores the importance of cross-agency coordination, transparency, and adaptive risk management, aligning closely with the governance challenges identified in this review [5].

However, in spite of these long-standing structures, there is imbalance in the way biosafety is governed in the various institutions. The mechanisms of oversight are quite different in terms of composition, the scope of power and the ability to provide. Fragmented oversight is due to differences in the interpretation of regulations, lack of consistency in interagency communication, and inconsistent compliance with federal guidance. In addition, the current systems tend to be reactive and not preventive, focusing on verification of compliance and not constant improvement. This compliance-oriented orientation denies proactive risk management and coordination of response as well as performance evaluation. The issue of standardized measures of responsibility or formal governance systems also adds to the problem of maintaining consistent levels of biosafety across the national research environment [6].

Project-Management Principles Relevant to Biosafety

There are established methods to enhance accountability, handle complex risks, and create consistent performance in high-stakes settings given the provisions of the project-management governance frameworks, such as the PMBOK, PRINCE2, and ISO 21500. These frameworks highlight the significance of defining the roles, stakeholders’ involvement, systematic risk analysis, and performing of the measurable performance indicators. These principles can bring rigor and transparency to processes of oversight where there is generally informality and inconsistency when adapted to biosafety [7].

Although several of the project-management sources cited originate from sectors such as construction and infrastructure development, they are included here to illustrate transferable governance mechanisms such as role clarity, stakeholder mapping, and performance measurement that are directly applicable to biosafety oversight in high-risk research environments.

Project-management governance might be applicable in the context of biosafety to guarantee that responsibilities are clearly placed, the pathways of decision-making processes are clear, and communication lines are harmonious. The project-style risk register may allow institutions to recognize, prioritize, and track hazards on an on-the-fly basis and performance indicators would offer a quantifiable way of safety outcomes phenomena. A combination of these tools would help not only to enhance efficiency in operations but also to be more accountable at the institutional and federal levels [8]. The most important principle of project-management governance and its implementation in terms of biosafety oversight is summarized in Table 1.

**Table 1:** Integration of project-management governance principles into biosafety oversight functions This table summarizes key project-management governance principles adapted from internationally recognized frameworks such as PMBOK, PRINCE2, and ISO 21500, illustrating how each principle can enhance biosafety oversight in high-risk biological research facilities. The integration of these governance elements supports structured accountability, transparent communication, and data-informed decision-making throughout all levels of biosafety governance. Table created by the authors using conceptual synthesis derived from the project-management and biosafety governance literature. PMBOK: Project Management Body of Knowledge

Project-Management Governance Principle	Description (Based on PMBOK, PRINCE2, ISO 21500)	Application to Biosafety Oversight
Defined accountability and role clarity	Establishes explicit responsibility matrices to ensure every task and decision has an identified owner.	Clarifies oversight responsibilities among federal agencies, institutional leadership, biosafety committees, and laboratory managers [9]
Stakeholder mapping and engagement	Systematically identifies all relevant stakeholders and maintains active communication channels throughout the project life cycle	Promotes collaboration between regulators, institutional biosafety officers, researchers, and facility management to align safety objectives [10]
Risk register and proactive mitigation	Documents, assesses, and monitors risks through a living register that guides mitigation actions and contingency planning	Enables continuous monitoring of biosafety hazards, prioritization of control measures, and transparent reporting of incidents [11]
Performance metrics and key indicators	Uses measurable outcomes to evaluate progress, quality, and compliance with project objectives	Introduces quantifiable indicators for biosafety performance, compliance, and culture improvement across laboratories [12]
Continuous monitoring and feedback loops	Incorporates regular review cycles, audits, and lessons learned for ongoing improvement	Strengthens institutional learning, supports data-driven policy updates, and enhances national oversight consistency [13]
Change management and adaptability	Provides structured processes for responding to new risks, technologies, or policy shifts	Ensures that biosafety programs evolve with emerging research methods and regulatory requirements [14]

Bringing Governance into Biosafety

The integration of project-management governance into biosafety oversight allows institutions to shift from compliance-based monitoring toward proactive risk management. This approach also improves accountability, inter-agency coordination, and continuous performance evaluation [15].

In the framework of biosafety programs, this integration can be conducted in the form of formal governance boards/committees that would review the biosafety performance, resource allocation, and compliance data probing. These constructs would create periodic performance reviewing cycles where the institution would be in a position to know the weaknesses and take corrective measures as well as keeping track of the results in a continuous feedback mechanism [16]. The plausibility of this approach can be supported using the lessons of the other high-risk industries. Self-governing boards and independent reviewer panels are beneficial in nuclear energy so that redundancy and accountability are guaranteed whereas the standardized audit mechanism and incident reporting systems in aviation allow the learning of near-misses. Likewise, information security governance can be seen as project-based controls of the vulnerability of the systems through the anticipation and mitigation of vulnerabilities [17]. The similar benefits in accountability, transparency and resiliency may be obtained through the similar models applied to biosafety oversight.

Proposed Governance Model

This review suggests a three-layer project-management governance framework, which is aimed at enhancing biosafety regulation, and the framework is based on the conceptualization of thematic synthesis of biosafety and project-management literature. The model establishes strategic, operational and project level roles, to create vertical congruency and responsibility in institutions [18]. The federal agencies (CDC, the NIH, and the USDA) at the strategic level link the objectives to the national biosafety, standardized performance indicators, and cross-agency communication of information. Flowing down to the working level, the federal goals are converted into organizational policies by the institutional leadership and biosafety committees, which also distribute resources and monitor internal auditing mechanisms through the organized governance boards [19]. These policies are enforced at the project level by laboratory managers and the biosafety officers in the daily operations, keep risk registers and report performance of the project to the governing levels.

**Figure 1:**
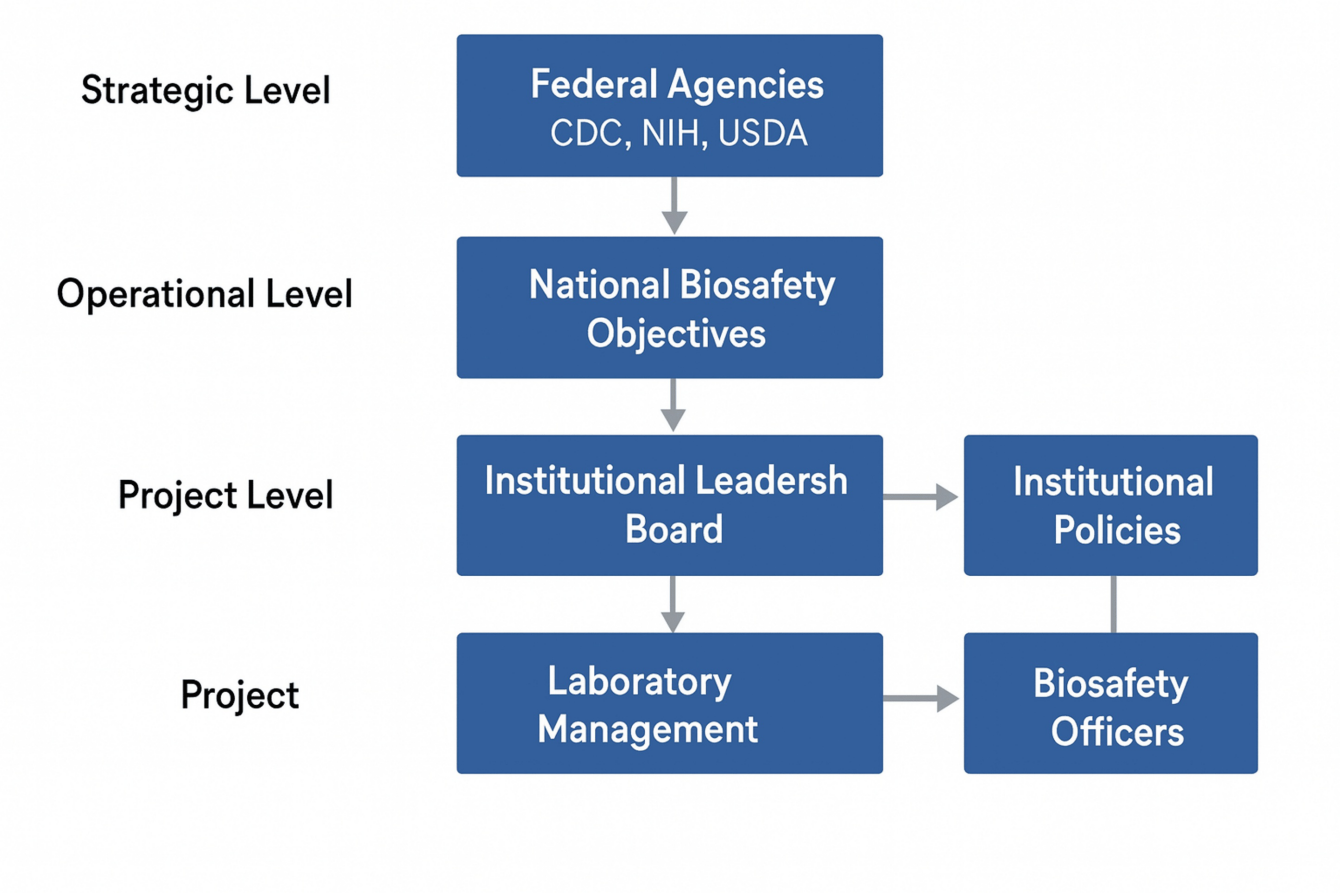
Project-management governance model for biosafety oversight in the United States Figure was created by the authors using conceptual synthesis derived from the reviewed literature. CDC: Centers of Disease Control and Prevention; NIH: National Institutes of Health; USDA: United Stated Department of Agriculture

The model is based on the use of continuous feedback loops, which are used to tie the operational and project activities with strategic oversight. These loops enable meeting the national policy on compliance trends and incidents as well as performance measures information in order to influence the improvement plans at the institutional level. The model helps maintain flexible governance that can adjust to emerging biosafety risks and technological innovations by instilling the elements of accountability, performance appraisal and continuous learning [20]. The schematic representation of this suggested governance model is shown in Figure 1.

Figure 1 illustrates a three-tier project-management governance structure for strengthening biosafety oversight in high-risk biological research institutions, comprising strategic (federal agencies), operational (institutional leadership and biosafety committees), and project-level (laboratory management and biosafety officers) controls, linked through continuous feedback loops. The level of strategy involves the federal agencies that also draw the national biosafety goals, performance measures, and interagency communication, including CDC, NIH, and USDA. The institutional leadership with the biosafety committees form the operational level, which converts national goals into institutional policies, governance procedures and strategies of allocating resources. In the project level, there are laboratory management and biosafety officers that perform day-to-day implementation, risk follow up and performance reports. The three levels have feedback loops that allow constant learning, responsibility, and responsive excellence in the national biosafety oversight system.

This three-level project-management governance scheme depicts strategic, operational, and project level to enhance biosafety as a strategic approach of high-risk bio-research centers in the United States.

Implementation Considerations

Applying the proposed model of governance will have to be fitted with the already existing policies, namely the NIH Guidelines, the Federal Select Agent Program, and institutional biosafety management systems. The model, instead of substituting the existing regulatory frameworks, is designed to reinforce them by naming it and bringing sense of structure and operations. The success of it is to a great extent connected with organizational culture and the participation of leaders. Good institutional belief in safety, openness, and investing towards training are requisite to facilitate the improvement of governance practices successfully into biosafety operations [6].

However, real-life issues are expected. Small institutions will have access to little financial or human resources to have in place elaborate governance boards or performance monitoring systems. The overlapping of federal and state regulatory bodies may also be a problem that will lead to difficulty in coordination and information sharing. A gradual implementation plan is adviser to overcome these issues and pilot programs in a small sample of institutions should be introduced first to prove their feasibility and perfect their operations before going national.

Future Directions

The proposed governance model should be improved by further research on how the proposed model could be validated by empirical studies. BSL-3 and BSL-4 pilot implementations would offer good information regarding its effects on the compliance outcomes, incident rate, and its involvement with its stakeholders. Inter-institutional comparisons would be able to determine the best practices and obstacles to the adoption. Simulation research can be helpful to test the relationship between the maturity of governance and biosafety performance and institutional resilience as well.

Besides the empirical validation, the cooperation with the industries utilizing the developed systems of governance, including nuclear energy, aviation, or information technology, may bring even more information. Creating quantifiable measures of governance performance and creating a national biosafety performance data base would be used to benchmark and to continuously enhance. Such initiatives may over the long run help in establishing a common, evidence-based way of governing biosafety in the United States [20].

## Conclusions

Fragmentation, disjointed efforts, and the lack of proactive risk management remain enduring challenges in United States biosafety regulation. This review proposes that applying project-management governance principles can address these deficiencies by creating a systematic, transparent, and accountable framework that enhances coordination and responsiveness across agencies and institutions. By defining roles and establishing measurable performance indicators, biosafety management can evolve beyond regulatory compliance. Formalized feedback mechanisms across strategic, operational, and project levels further support governance, leadership, and continuous improvement. Such an integrated approach promotes balanced regulatory oversight, aligns operational practices with national biosafety objectives, and fosters long-term collaboration among federal agencies, research institutions, and biosafety professionals, ensuring that the United States maintains the highest standards of safety, integrity, and responsibility in high-risk biological research.
